# A246 EXPLORING THE ROLE OF LUMINAL PROTEASES IN NOCICEPTION MODULATION FROM PATIENTS WITH ACTIVE AND REMISSIVE INFLAMMATORY BOWEL DISEASE

**DOI:** 10.1093/jcag/gwae059.246

**Published:** 2025-02-10

**Authors:** H M Wood, K Blazkova, F F Faucher, P M Sheth, S J Vanner, D E Reed, M Bogyo, A E Lomax

**Affiliations:** DBMS, Queen’s University, Kingston, ON, Canada; Stanford University, Stanford, CA; Stanford University, Stanford, CA; DBMS, Queen’s University, Kingston, ON, Canada; DBMS, Queen’s University, Kingston, ON, Canada; DBMS, Queen’s University, Kingston, ON, Canada; Stanford University, Stanford, CA; DBMS, Queen’s University, Kingston, ON, Canada

## Abstract

**Background:**

Abdominal pain poses a significant challenge for individuals with inflammatory bowel disease (IBD). Despite current treatments that target inflammation, IBD-associated abdominal pain often persists even in the absence of inflammation, negatively impacting patients’ quality of life. This persistence suggests that factors other than inflammation may be at play. Our previous research suggests that bacterial proteases can directly influence the excitability of dorsal root ganglia neurons, many of which are pain-sensing. Building on this, we hypothesize that proteases, both of host and bacterial origin, play a role in pain modulation during the active and remission phases of IBD.

**Aims:**

Determine whether luminal proteases in IBD fecal samples induce changes in pain signalling.

**Methods:**

The effects of fecal supernatants (FS) from patients with active or remissive IBD and healthy volunteers (HV), on pain-sensing neurons were assessed using *ex-vivo* single-unit afferent nerve recordings from mouse colons. A protease inhibitor cocktail (PIC; 1:1000) and a protease-activated receptor (PAR)-2 antagonist (GB83; 10µM) were independently applied in the bioassay to determine whether these inhibited the excitatory effect of the FS. In addition, the participant FS were tested for proteolytic cleavage of the N-terminal domain of protease-activated receptor PAR2 using a novel enzymatic assay.

**Results:**

FS from HV [N=5] had no effect on afferent nerve excitability (p>0.05). FS from active IBD patients [N=15] increased action potential discharge from colonic afferent nerves by 85% (p<0.0001) and selectively increased the activation of high-threshold units, which are putative nociceptors, by 44% (p<0.01). A protease inhibitor cocktail and PAR2 antagonist both independently inhibited the excitatory effects of IBD FS (p>0.05) on afferent nerve activity. In contrast, FS from IBD patients in remission [N=15] did not excite colonic afferent nerves (p>0.05). Interestingly, these findings were found to be consistent when IBD was split into disease subtypes: Crohn’s disease and ulcerative colitis. Furthermore, when normalized to total protein content, active disease yielded significantly greater PAR2 cleavage activity (p<0.05), while the remission samples were not significantly different than the HV (p>0.05). This PAR2 cleavage activity was found to be correlated to neuronal excitation (R^2^=0.4721, p<0.001).

**Conclusions:**

Our findings suggest that active IBD leads to the generation of luminal mediators, including proteases acting on PAR2, that activate visceral nociceptive neurons. These luminal mediators are less abundant when inflammation is in remission. These data suggest that targeting proteases could offer a promising therapeutic approach for pain management in IBD.

**Funding Agencies:**

CIHR

